# Testosterone-Induced Heart Failure in a Professional Dancer

**DOI:** 10.1016/j.jaccas.2026.107278

**Published:** 2026-03-25

**Authors:** Kyle Udd-Garnica, Tyler Morad, Martine Webb, Jeffrey J. Hsu

**Affiliations:** a Division of Cardiology, Department of Medicine, University of California, Los Angeles, California, USA; b Department of Medicine, University of California-Los Angeles, Los Angeles, California, USA; c Cardiovascular Division, Department of Medicine, University of California Irvine, Irvine, California, USA; d Division of Cardiology, Greater Los Angeles VA Medical Center, Los Angeles, California, USA

**Keywords:** awareness, cardiomyopathy, drug abuse, exercise, reduced ejection fraction

## Abstract

**Background:**

Recreational anabolic-androgenic steroid (AAS) use is common among noncompetitive adults and is strongly associated with increased cardiovascular risk.

**Case Summary:**

A 34-year-old professional dancer presented with 1 month of progressive dyspnea on exertion and orthopnea in the setting of testosterone use. Transthoracic echocardiogram revealed a left ventricular (LV) ejection fraction of 16%, global LV hypokinesis, and LV dilation. He was diagnosed with nonischemic cardiomyopathy, initiated on diuresis and guideline-directed medical therapy, and counseled on avoidance of testosterone.

**Discussion:**

AAS users have a significantly higher incidence of acute myocardial infarction, coronary intervention, venous thromboembolism, arrhythmia, and heart failure compared with matched control patients. Although some markers of cardiac function recover after cessation, others remain impaired.

**Take-Home Messages:**

AAS use is common among adult recreational athletes and is an underrecognized cause of LV dysfunction and heart failure. Early recognition, diagnosis, and cessation of AAS can significantly improve cardiac function and at least partially reverse AAS-associated cardiotoxicities.

## Graphical Abstract

**Figure:**
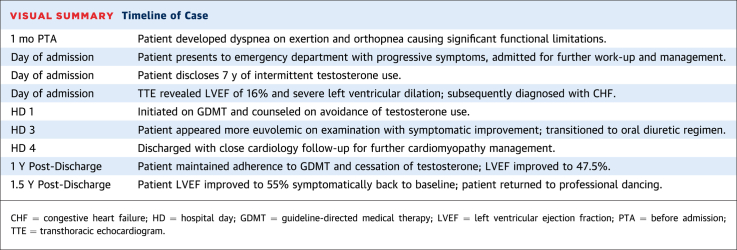

## History of Presentation

A 34-year-old man presented with progressive shortness of breath on exertion and while lying flat. He is currently a professional dancer, actor, and model, and a former collegiate track and field athlete. He typically exercises 6 d/wk for multiple hours per day, including 2 to 3 hours of strength training and 1 hour of endurance training per session. On presentation, he reported 1 month of progressive dyspnea even when walking up a few steps. He denied associated chest pain, palpitations, or cough. He has no family history of cardiac disease. He endorsed social alcohol use and intermittent supplemental testosterone use for 7 years. Details regarding the dose and frequency of supplemental testosterone were not available.

**Take-Home Messages**

• AAS use is increasingly common among adult recreational athletes and is an underrecognized cause of LV dysfunction and heart failure.
• The cardiotoxicities associated with AAS are synergistic, dose-dependent, and can be at least partially reversible.
• Early recognition and cessation of AAS along with appropriate medical therapy can significantly improve cardiac function.

Physical examination revealed a muscular, 6-ft 4-in male in no acute distress. Lung examination revealed clear lung fields bilaterally without crackles or wheezes. Cardiac examination revealed a tachycardic rate with regular rhythm and normal S_1_ and S_2_ without murmurs, rubs, or gallops. Jugular venous pressure was noted to be 12 cm H_2_O. No edema was noted in his lower extremities.

## Past Medical History

The patient's past medical history was unremarkable. He had no personal or family history of cardiovascular disease or sudden cardiac arrest or death.

## Differential Diagnosis

Differential diagnosis included new-onset congestive heart failure, acute coronary syndrome, tachyarrhythmia such as a supraventricular tachycardia, valvular disease such as aortic stenosis, pneumothorax, pulmonary hypertension, and anemia.

## Investigations

On presentation, basic metabolic panel was significant for elevated creatinine. Complete blood count was unremarkable. High-sensitivity troponin was 17 ng/L with a repeat of 18 ng/L (reference range: <5 ng/L), and B-type natriuretic peptide was 755 pg/mL (reference range: <100 pg/mL). Electrocardiogram demonstrated sinus tachycardia with right axis deviation, inferolateral ST-segment depressions, and left ventricular (LV) hypertrophy. A transthoracic echocardiogram revealed a left ventricular ejection fraction (LVEF) of 16% (Video 1), global LV hypokinesis, severe LV dilation (LV end diastolic diameter: 7.7 cm), normal right ventricular systolic function, normal pulmonary artery systolic pressure, and moderately elevated right atrial pressure. Chest radiograph was notable for an enlarged cardiomediastinal silhouette, central vascular prominence, and mild bronchial wall thickening without focal consolidation, pleural effusions, or pneumothorax.

**Figure 1:**
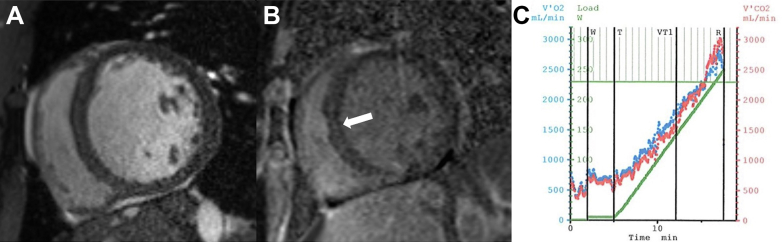
Initial Diagnostic Studies (A and B) Cardiac magnetic resonance was obtained 3 days postindex hospitalization. (A) Short-axis view of the basal left ventricle demonstrated evidence of severe left ventricular dilation. (B) Phase sensitive inversion recovery sequence images at the same level revealed linear, mid-wall, delayed enhancement of the interventricular septum (arrow). (C) On cycle ergometer cardiopulmonary exercise testing, VO2 was 25.6 mL/kg/min (72% predicted) with peak power of 248 W. VO2 = peak oxygen uptake.

Pharmacologic stress myocardial perfusion imaging performed during index hospitalization was negative for ischemia. Cardiac magnetic resonance obtained 3 days post-index hospitalization was notable for LV dilation and delayed gadolinium enhancement in the mid-wall of the interventricular septum (Figures 1A and 1B). LVEF was quantified at 19%, LV myocardial mass was 323 g, LV end-diastolic volume was 545 mL, and right ventricular end-diastolic volume was 258 mL. Cardiopulmonary exercise testing performed 2 months post-index hospitalization revealed a peak oxygen uptake of 25.6 mL/kg/min (72% of predicted value) and peak power of 248 W (Figure 1C). Genetic testing revealed a negative Invitae cardiomyopathy panel. Additional laboratory evaluation was notable for a thyroid stimulating hormone, hemoglobin A1c, and lipid panel all within normal limits other than an high-density lipoprotein of 22 mg/dL (reference range: >40 mg/dL). Ferritin was 388 ng/mL (reference range: 8-350 ng/mL), and iron and thiamine levels were within normal limits. Hepatitis C antibody was reactive with a resultant negative hepatitis C RNA polymerase chain reaction. Phosphatidylethanol testing did not reveal evidence of severe alcohol consumption history.

## Management (Medical/Interventions)

Given the patient's presenting symptoms, examination findings, and diagnostic evaluation, he was diagnosed with congestive heart failure with reduced ejection fraction secondary to nonischemic cardiomyopathy in the setting of testosterone use. Cardiology was consulted, and the patient was initiated on intravenous diuretic therapy and guideline-directed medical therapy (GDMT) with beta blocker, angiotensin receptor-neprilysin inhibitor, sodium-glucose cotransporter-2 inhibitor, and mineralocorticoid receptor antagonist. He was counseled to avoid testosterone and other performance-enhancing drugs. The patient appeared euvolemic after aggressive diuresis with associated symptomatic improvement, and he was discharged with close cardiology follow-up for further cardiomyopathy management and endocrinology follow-up for management of secondary hypogonadism secondary to testosterone use.

## Outcome and Follow-Up

After over a year of target-dose GDMT and cessation of supplemental testosterone use, the patient's LVEF improved to 55% (Video 2), with normalization of his LV end-diastolic diameter (5.7 cm). He was started on clomiphene by endocrinology with significant improvement in energy levels and overall well-being. The patient has since returned to dancing and acting without cardiovascular limitations.

## Discussion

We present a case of a highly active adult male athlete with intermittent use of exogenous anabolic-androgenic steroid (AAS) resulting in severe LV dysfunction, volume overload, and heart failure.

Recreational AAS use is common among noncompetitive adults, with estimates suggesting that 2.9 to 4.0 million US adults have used AAS at some point in their lives.^1^ Importantly, most AAS users are not competitive athletes—approximately 70% to 78% are recreational exercisers or individuals using these drugs primarily for cosmetic purposes.^2^ Off-label testosterone use in men with normal testosterone levels has become more common,^1^ with online and direct-to-consumer marketing likely playing a role in this trend. Although little is known about actual administration schedules or the dosing protocols used, typical dose ranges for AAS have been published.^2^ However, supraphysiological levels of testosterone are often desired to facilitate muscle growth.^1^^,^^3^ Such AAS use among adults who are not competitive athletes is strongly associated with increased cardiovascular risk, including both structural and functional cardiac abnormalities, adverse vascular changes, and atherogenic lipid profiles.^4^

Large cohort and cross-sectional studies show that AAS users have a significantly higher incidence of acute myocardial infarction (adjusted HR [aHR]: 3.00), coronary intervention (aHR: 2.95), venous thromboembolism (aHR: 2.42), arrhythmia (aHR: 2.26), cardiomyopathy (aHR: 8.90), and heart failure (aHR: 3.63) compared with matched control patients, with these risks persisting over more than a decade of follow-up.^5^ Echocardiography and additional imaging studies in recreational AAS users consistently demonstrate an increased risk of reduced LV systolic and diastolic function, increased LV mass, and accelerated coronary atherosclerosis, with the degree of dysfunction correlating with cumulative AAS exposure.^4^^,^^5^

AAS is often an underrecognized culprit for cardiac disease, and a multitude of factors including the lack of physician awareness, social taboo around admitting to steroid use, and periodic cycles of taking the medication decrease the likelihood of reporting AAS use and, ultimately, diagnosing AAS-related cardiomyopathy. The cardiotoxicity of AAS arises from a combination of direct myocardial injury, adverse remodeling, vascular dysfunction, inflammation, dyslipidemia, and prothrombotic effects, all of which synergistically increase the risk of heart failure, arrhythmias, and premature cardiovascular events.^4^, ^5^, ^6^

Some cardiovascular changes may persist after cessation of AAS, and sudden cardiac events have been reported even in young, otherwise healthy athletes.^3^ The American College of Sports Medicine, American College of Cardiology, and American Heart Association recognize AAS abuse as a modifiable cardiovascular risk factor and recommend early screening and risk stratification in this population.^7^

LVEF appears to recover after cessation, with one study showing that past users (median: 300 days since last use) had LVEF comparable with nonusers (mean: 58% vs 58%), whereas current users had significantly lower LVEF (51%). However, more sensitive markers of systolic dysfunction, particularly LV global longitudinal strain, remain impaired years after cessation. Past users demonstrated persistently reduced LV global longitudinal strain (−16.7%) compared with nonusers (−18.2%), despite being off AAS for a median of 30 months. LV mass appears to decrease after cessation, with past users showing LV mass comparable with nonusers, whereas current users had significantly elevated LV mass.^8^ In this case, after initiation of GDMT and cessation of AAS, there was a notable improvement in LV size and systolic function.

Arterial stiffness and microvascular dysfunction appear to persist after AAS cessation. Past users continue to show reduced arterial elasticity, increased pulse wave velocity, reduced carotid artery compliance, and increased carotid intima-media thickness compared with nonusers.^9^ Most concerningly, coronary microvascular dysfunction, measured as reduced myocardial flow reserve, persists years after AAS discontinuation. This impaired myocardial flow reserve was independently associated with longer cumulative duration of AAS use, suggesting a dose-dependent effect that may not fully reverse.^10^ To date, there has been no suggestion of anginal symptoms for this patient; however, regular cardiology follow-up and vigilance about the possibility of early onset atherosclerosis have been recommended given his prior AAS use.

Per the 2025 American College of Cardiology/American Heart Association scientific statement for competitive athletes with cardiovascular disease,^7^ which may also be applied to high-level recreational athletes who engage in vigorous exercise, return to sport or vigorous activity after a diagnosis of dilated cardiomyopathy is reasonable after comprehensive risk stratification and a shared decision-making discussion with the athlete. Although there is no specific protocol recommended to determine an individual athlete's candidacy for returning, higher-risk features for ventricular arrhythmias (eg, reduced LVEF, symptoms, high scar burden on cardiac magnetic resonance) are important factors to include in the shared decision-making discussion and in continued reassessments. Furthermore, GDMT should be optimized in these athletes before their return to sport.

## Conclusions

This case demonstrates cardiotoxicity as one of the life-threatening dangers associated with AAS use among recreational adult athletes. With early recognition and cessation of AAS, some of these effects appear to be reversible, highlighting the importance of screening for AAS use in athletes, and consideration of AAS use as a possible cause of new-onset cardiac dysfunction.

## Funding Support and Author Disclosures

The authors have reported that they have no relationships relevant to the contents of this paper to disclose.

## Appendix

Video 1 Transthoracic Echocardiogram in the Apical 4-Chamber View Acquired at the Time of Hospital Admission Demonstrating Severely Reduced Left Ventricular Ejection Fraction (16% by Simpson's Biplane Method) and Severely Increased Left Ventricular Cavity Size

Video 2 Transthoracic Echocardiogram in the Apical 4-Chamber View Acquired at Clinic Visit 1.5 Years After Initial Presentation and After Cessation of Anabolic-Androgenic Steroids Demonstrating Improved Left Ventricular Ejection Fraction (55% by Simpson's Biplane Method) and Normalized Left Ventricular Cavity Size
